# A Displacement Field Perception Method for Component Digital Twin in Aircraft Assembly

**DOI:** 10.3390/s20185161

**Published:** 2020-09-10

**Authors:** Bing Liang, Wei Liu, Kun Liu, Mengde Zhou, Yang Zhang, Zhenyuan Jia

**Affiliations:** School of Mechanical Engineering, Dalian University of Technology, Dalian 116024, China; liangbing2016@mail.dlut.edu.cn (B.L.); dgjrlk_2013008@mail.dlut.edu.cn (K.L.); mengde@mail.dlut.edu.cn (M.Z.); zy2018@dlut.edu.cn (Y.Z.); jzyxy@dlut.edu.cn (Z.J.)

**Keywords:** aircraft manufacture, assembly, full-field displacement perception, digital twin, matrix completion

## Abstract

Full-field displacement perception and digital twins for core components play a crucial role in the precision manufacturing industry, such as aviation manufacturing. This paper presents a real-time full-field displacement perception method for the combination of online multipoint displacement monitoring and matrix completion theory. Firstly, a conceptual full-field displacement perception model based on the observed information of the multi-points is established. To obtain the full-field displacements of a core component, the component is divided into plentiful discrete points, including observed and unobserved points, based on which the relationship between the observed points and the full-field displacements is established. Then, the solution method of the full-field displacement perception model is proposed. Based on the matrix completion principle and the big data of the simulation, the optimization problem is employed to work out the model and, meanwhile, the pseudo-code is put forward. Finally, the full-field displacement perception experiments are performed. Repeated experiments show that the max error of the displacements calculated by the proposed method can be less than 0.094 mm and the median error can be less than 0.054 mm, while the average time frame can be less than 0.48 s, which is promising considering the high precision and efficiency requirements of the assembly of large aircraft.

## 1. Introduction

Real-time three-dimensional displacement monitoring is the key to ensure the smooth progress of precision manufacturing, thus the full-field displacement perception and digital twin for some core components are of great importance. For example, in the process of aircraft assembly, since the assembly quality of the aircraft strongly depends on the position accuracy of the positioners on the assembly tooling, the full-field displacements of the positioners need to be monitored. However, in most practical applications, due to the limitation of the monitoring space and the equipment, etc., the acquired information is insufficient and discontinuous, which brings great difficulties to the perception of the full-field displacements. With the development of sensing technology and information theory, high-efficiency calculation with big data based on high-precision sensing information provides a new and effective way to obtain the full-field displacements in digital twin applications.

### 1.1. Displacement Sensing Technology

In the sensing technology of the displacement field, high-precision monitoring methods have been adopted in the displacement inspection field, such as laser ranging methods, electromagnetic ranging methods, photogrammetric methods and multi-sensor methods. Thus, in the process of aircraft assembly, high-precision measuring equipment, such as laser trackers, proximity sensors and industrial photogrammetry are commonly used to acquire the assembly process data. Laser trackers [1,2,3] are widely used in large-scale coordinate measurements, with the measurement range covering 10–60 m and the precision reaching ±(15 μm + 6 μm/m). Inductive proximity transducers [4,5,6] are widely used in high-precision displacement inspection fields with the advantages of compact volume, high precision (in micron scale) and fast response. For highly precise and fast shape measurement, industrial photogrammetry [7,8,9], with the measurement characteristics of both a high precision of up to ±(8 μm + 8 μm/m) and high efficiency, is more commonly used.

Meanwhile, many studies on displacement field perception have been carried out by scholars in various fields. An et al. [10] developed an omnidirectional 3D laser ranging system to realize 3D reconstruction in indoor and outdoor scenes based on a 2D laser rangefinder and a camera. The system was proved to have a good performance in the actual tests with an average error of 0.9875 pixels. Kim et al. [11,12] proposed a novel noncontact displacement field measurement system consisting of a camera and two laser sensors. Performance validation experiments were conducted in a measurement range of 30 m with minimum accuracies of 4 mm and 0.5° and precisions of 0.7 mm and 0.3° for the 6-DOF motions. Liu et al. [13] presented a 3D sensing system to facilitate the high-precision assembly of two components. The system consists of three orthogonal microscopic cameras. Accuracy tests were conducted, and the alignment errors were less than ±2 μm in terms of position and ±0.1° in terms of orientation based on the proposed system. Based on a laser line projector and a camera, Palomer et al. [14] presented a laser scanner for underwater 3D perception. After calibration of the proposed laser scanner, the error was less than 1 mm in the 0.5 m~1.2 m range. Through the actual tests, the measurement average errors were proved to be 0.44 mm and 0.98 mm with a standard deviation of 0.35 mm and 0.72 mm in air and underwater, respectively. Lembono et al. [15] presented a 3D measurement system based on a laser range finder (LRF) and a robot, as well as a calibration method to reduce the mean planar errors. Experiments based on the system were conducted and the proved accuracy was 0.23 mm using inexpensive and convenient means. To achieve the high-precision measurement of large components, Masahiro et al. [16] developed an on-machine system based on a laser tracker and a touch-trigger probe. The diameter of a steam turbine rotor was measured by the system with an uncertainty of only 3.4 μm.

### 1.2. Processing Approaches for Displacement Field

In the aircraft assembly process, it is of great importance to obtain the full-field displacements of some core components. However, complex working conditions, such as the compact measurement space, will bring great difficulties for the sensing equipment to achieve the full-field displacement measurement. Thus, a lot of research on the processing approaches, such as data mining algorithms, matrix completion algorithms, and modal analysis algorithms, has been carried out to facilitate the prediction or estimation of the unobservable values based on limited observations.

Li et al. [17] proposed a data-driven approach to design the measurement matrix and a support recovery method for complex sparse signals. Numerical results showed that the proposed approach can achieve sparse support recovery with low computational complexity and better performance with much less computation time. Li et al. [18] built a versatile probabilistic model for diagnosis and prognosis based on the concept of a dynamic Bayesian network. To illustrate the proposed method, the experiments were conducted on crack opening displacement tracking in an aircraft wing fatigue crack growth example. Knapp et al. [19] proposed an ellipsoidal function to establish a model of the directed energy deposition process based on the success of parabolic function fitting to 2D clad surfaces. To predict the bead geometry in robotic gas metal arc welding for rapid manufacturing, Xiong et al. [20] proposed a novel method based on the application of a neural network and a second-order regression analysis. For inner dimension measurements, Li et al. [21] proposed a high-speed, in situ measuring method based on revolving double laser beams. By revolving the two laser beams consecutively, measured data samples were collected. Then, inner dimensions were extracted from the statistical characteristics through fitting the measured data. Zambal et al. [22] proposed a manufacturing database that creates a digital twin of the part as it is manufactured. A 3D surface model of the current layer is generated by continuously moving the optical sensors across the surface. Thus, it can facilitate better decisions about the mechanical properties of the part and consequently the need for re-work. Lee et al. [23,24] proposed a displacement field reconstruction method based on modal analysis with a distributed plate model for predicting continuous deformations of a flexible thin-wall workpiece. Application tests on compressor disk machining illustrated that the displacement field of the annular plate can be reconstructed with relatively high precision.

### 1.3. Displacement Field Perception for Digital Twin

Digital twin is one of the most promising enabling technologies for realizing smart manufacturing. Digital twins are characterized by the seamless integration between the cyber and physical spaces (including the physical model, the sensor update and the historical data) [25]. In the precise manufacturing process, due to the importance of the position accuracy of the core components, a digital twin for the components is necessary to view the change in the displacement field in real time.

DebRoy et al. [26] provided a perspective of the current status and research needs for the main building blocks of a first-generation digital twin for additive manufacturing. Meanwhile, the paper pointed out that, considering the effect on the properties by the manufactured components caused by the geometry, finite element methods (FEMs) and big data methods are starting to be utilized for the calculations of residual stresses and displacements. Mandolla et al. [27] proposed a digital twin for additive manufacturing in the aircraft industry through the exploitation of block chain solutions. Taking a blade compressor as an example, a 3D modeler software was adopted to generate the 3D model and, meanwhile, a rapid prototyping assistant software was employed to display in real-time the spatial position of the components. Cerrone et al. [28] proposed a digital twin of as-manufactured specimens based on the finite element model, which can resolve the ambiguity of the crack opening displacement based on the as-manufactured geometry, instead of a distribution of possible specimen geometries or nominal geometry. Tabar et al. [29] proposed a method for the identification and sequence optimization of the geometry weld points within a geometry assurance digital twin, which can ensure that the geometrical quality of the assemblies are within the acceptable error ranges. Applying the concept of digital twins in production processes, Aderiani et al. [30] developed a selective assembly technique for sheet metal assemblies. The approach presented was applied to three industrial cases of sheet metal assemblies and led to a considerable reduction of the final geometrical variation and mean deviation.

### 1.4. Motivation of This Work

In the process of aircraft assembly, as the position accuracy of the core positioners on the assembly tooling are of great importance, high-accuracy and high-efficiency full-field displacement perception of the positioners is required.

The motivation of our work is to propose a real-time displacement field perception method with high precision and high efficiency based on displacement observation of a small amount of points, combining the matrix completion theory. Thus, the digital twin for core components can be achieved with the displacement field information.

The rest of this paper is organized as follows. Section 2 describes the full-field displacement modeling method based on the displacements of the observed points. Section 3 details the model solution process based on the matrix completion theory. In Section 4, displacement field perception experiments of the core positioners in the assembly process are presented. Finally, the paper is concluded in Section 5.

## 2. Modeling of the Full-Field Displacements

Forced assembly and vibrations are frequent occurrences in the assembly of large aircraft, because of the inevitable dimensional errors of the components, operator errors and drilling operations, which seriously affect the assembly accuracy. Assembly tooling is the base frame that supports the aircraft components and guarantees the position accuracy of the components. Thus, online position monitoring of the core positioners on the assembly tooling is indispensable.

**Figure 1:**
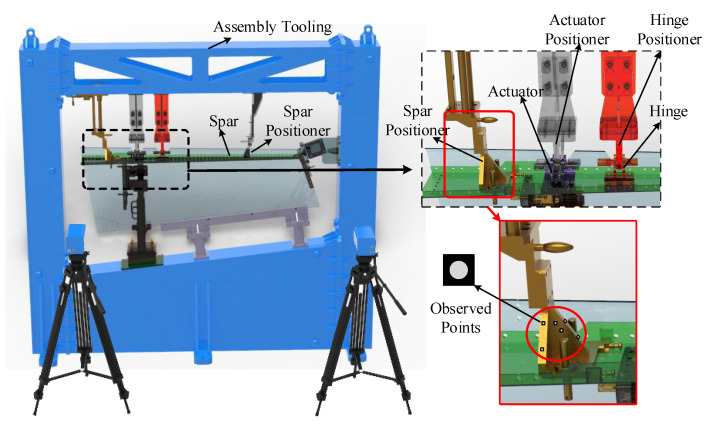
Monitoring process based on cameras.

However, only some points can be observed on the positioners, due to the compact space of the assembly tooling. A monitoring process based on cameras is shown in Figure 1.

**Figure 2:**
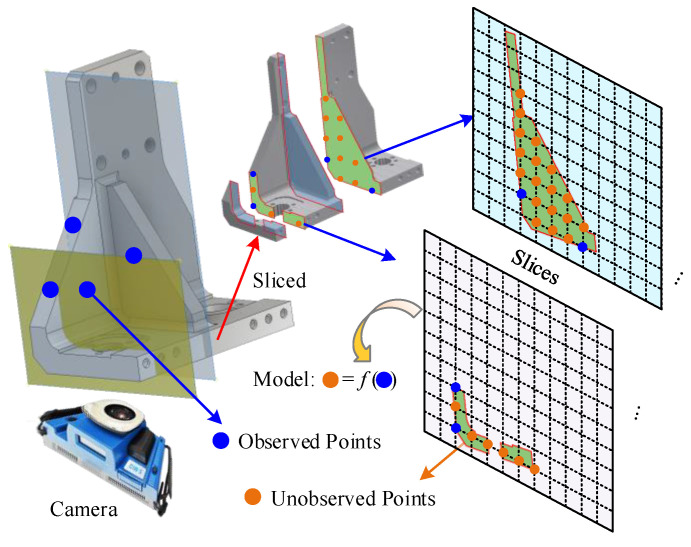
Measurement and modeling principle.

Thus, a position monitoring model based on the measurement information and structure relationship is proposed. Taking the spar positioner as an example, Figure 2 shows the measurement and modeling principle. Points on the spar positioner are observed by a camera. Meanwhile, in order to acquire the full-field displacements of the positioner under conditions of deformation, the positioner is sliced into pieces, on which the unobserved points are discretely located. Then, the model of the unobserved points is established based on the information of the observed points and the structural relationship among the discrete points on the positioner.

Supposing that the discrete points include both observed and unobserved ones, they are defined as:

(1) $$P=\left(\begin{array}{ccc} X & Y & Z \end{array}\right)$$

where $P\in {\mathbb{R}}^{N\times 3}$, $P_{\Omega }=\left(x\;y\;z\right)\in {\mathbb{R}}^{n_{1}\times 3}$ represents the projection of ***P*** in the observation space, namely the observed points and $P_{\Omega^{-1}}=\left(X\;Y\;Z\right)\in {\mathbb{R}}^{\left(N-n_{1}\right)\times 3}$ represents the projection of ***P*** outside the observation space, namely the unobserved points, thus

(2) $$P=\{\begin{array}{cc} P_{\Omega } & i,j\in \Omega \\ P_{\Omega^{-1}} & i,j\notin \Omega \end{array}$$

According to the structure relationship of the positioner, the following functional relationships can be expressed:

(3) $$P_{\Omega^{-1}}=f\left(P_{\Omega }\right)$$

Then, the following deformation relations are assumed:

(4) εP=F(P)

where ***εP*** represents the displacements occurring on ***P***, including both rigid and elastic displacements.

Thus,

(5) $$\varepsilon P_{\Omega^{-1}}=F\left(P_{\Omega^{-1}}\right)$$

(6) $$\varepsilon P_{\Omega }=F\left(P_{\Omega }\right)$$

Suppose that the deformation relations *F* can be solved as:

(7) $$F=sol\left(\varepsilon P_{\Omega }/P_{\Omega }\right)$$

Finally, combined with Equations (3), (5) and (7), the position monitoring model is established based on the measurement information and structure relationship:

(8) $$\varepsilon P_{\Omega^{-1}}=sol\left(\varepsilon P_{\Omega }/P_{\Omega }\right)\circ f\left(P_{\Omega }\right)$$

which is simply expressed as:

(9) $$\varepsilon P_{\Omega^{-1}}=\Pi \left(\varepsilon P_{\Omega },P_{\Omega }\right)$$

Thus, for practical monitoring, it is necessary to calibrate the relationship between $\varepsilon P_{\Omega^{-1}}$, $\varepsilon P_{\Omega }$ and $P_{\Omega }$.

## 3. Model Solution

**Figure 3:**
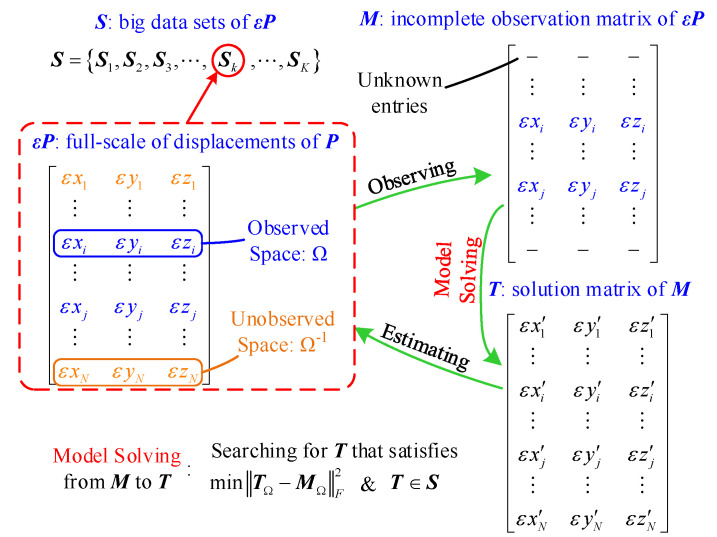
Principle of model solution.

The model solution strategy consists of three parts: transformation from the established model to an equivalent optimization problem, an optimization algorithm based on matrix completion and big data and pseudo-code of the optimization process. In the first part, the optimization target of the equivalent optimization problem is described, which satisfies the big data set based on the deformation simulation. In the second part, the optimization algorithm is described and, meanwhile, the step size, the stopping criterion and the initial value of the optimization process are suggested. Finally, in the third part, the pseudo-code is shown to facilitate the understanding of the algorithm. The principle of the model solution is shown in Figure 3.

### 3.1. Equivalent Optimization Problem

To transform the model problem into the optimization problem, the equivalent optimization target is necessary.

Let ***M*** be the incomplete observation matrix of εP:

(10) $$M_{ij}=\{\begin{array}{cc} \varepsilon P_{\Omega } & i,j\in \Omega \\ Unknown & i,j\notin \Omega \end{array}$$

In order to acquire the relationship between $\varepsilon P_{\Omega^{-1}}$, $\varepsilon P_{\Omega }$ and $P_{\Omega }$, simulations under various working conditions according to the production site are conducted. Thus, the big data set ***S*** is acquired, where $S_{i}\in {\mathbb{R}}^{N\times 3}$,i=1,2,⋯,K.

Firstly, two basic assumptions are put forward.

a. There are no observation errors with ***M***.

b. The sample size of ***S*** is large enough to cover the practical monitoring data εP, namely, εP∈S.

Therefore, under the above assumptions, there always exists a ‘k’ in i=1,2,⋯,K, which makes $\varepsilon P=S_{k}$.

Thus, the model-solving problem becomes an optimization problem [31,32,33,34]:

(11) $$\begin{array}{l} \min\;{\left\|T\right\|}_{*}\\ s.t.\;\;T\in S_{i},\;i=1,2,\cdots ,K\\ \;\;\;\;\;\;\;T_{\Omega }=M_{\Omega } \end{array}$$

where ***T*** is the intermediate quantity of εP in the optimization process and ${\left\|T\right\|}_{*}$ represents the nuclear norm of ***T***.

To remove the first assumption, the observation error ε is introduced.

Thus, the optimization problem becomes:

(12) $$\begin{array}{l} \min\;{\left\|T\right\|}_{*}\\ s.t.\;\;T\in S_{i},\;i=1,2,\cdots ,K\\ \;\;\;\;\;{\left\|M_{\Omega }-T_{\Omega }\right\|}_{F}^{2}<\varepsilon \end{array}$$

When removing the second assumption, data set ***S*** is still required to maintain a large number of samples, which will result in optimizing ***T*** in the range of ***S*** and greatly improving the optimization accuracy. Thus, the optimization problem finally becomes:

(13) $$\begin{array}{l} \min\;{\left\|T\right\|}_{*}\\ s.t.\;\;{\left\|M_{\Omega }-{\left(\sum_{i=1}^{K}\omega_{i}S_{i}\right)}_{\Omega }\right\|}_{F}^{2}<\varepsilon \end{array}$$

where

(14) $$\omega =\arg\min{\left\|\sum_{i=1}^{K}\omega_{i}S_{i}-T\right\|}_{F}^{2}$$

### 3.2. Solution of the Optimization Problem

To solve the above optimization problem, the singular value decomposition (SVD) approach is considered. The SVD of matrix ***T*** can be expressed as:

(15) $$T=U\Sigma V,\;\Sigma =diag\left(\left\{\sigma_{i}\right\}\right),\;1\leq i\leq rank(T)$$

where $\sigma_{i}$ are the positive singular values of ***T***. For each τ≥0, the soft-thresholding operator $D_{\tau }$ is defined as:

(16) $$D_{\tau }\left(T\right)=UD_{\tau }\left(\Sigma \right)V,\;D_{\tau }\left(\Sigma \right)=diag\left(\max\left\{\sigma_{i}-\tau ,0\right\}\right)$$

Thus, according to the singular value thresholding algorithm [32], the *h*th iteration operator of Equation (13) based on Equation (16) can be expressed as follows, which is proved to be convergent in [33].

(17) $$\begin{array}{l} T^{h}=D_{\tau }\left(Q^{h-1}\right)\\ Q^{h}=T^{h}+\delta_{h}\left(M_{\Omega }-{\left(\sum_{i=1}^{K}\omega_{i}^{h}S_{i}\right)}_{\Omega }\right) \end{array}$$

where

(18) $$\omega^{h}=\arg\min{\left\|\sum_{i=1}^{K}\omega_{i}^{h}S_{i}-T^{h}\right\|}_{F}^{2}$$

and a sequence $\left\{\delta_{h}\right\}$ of positive step sizes is introduced for the convenience of calculation.

Thus, the optimal solution of Equation (13) can be worked out by iterating Equation (17), where the step sizes, the stopping criterion, the parameter τ and the initial value should be determined in advance.

Many studies have been carried out on ways of selecting the step sizes. However, for simplicity, the undersampling ratio independent of the iteration count is employed, which is $\delta_{h}=\delta$ for h=1,2,⋯.

(19) $$\delta_{h}=\delta =\frac{valid\left(M\right)}{numel\left(\varepsilon P\right)}=\frac{numel\left(\varepsilon P_{\Omega }\right)}{numel\left(\varepsilon P\right)}=\frac{n_{1}}{N}$$

where valid(M) represents the available sampled entries of ***M*** and numel(εP) represents all sampled entries of ***εP***.

For the stopping criterion, a small constant tolerance *ξ* is considered, e.g., smaller than 10^−4^. Then the stopping criterion is suggested as:

(20) $$\frac{{\left\|\sum_{i=1}^{K}\omega_{i}^{h}S_{i}-T_{}^{h}\right\|}_{F}^{2}}{{\left\|\sum_{i=1}^{K}\omega_{i}^{h}S_{i}\right\|}_{F}^{2}}\leq \xi$$

Then, the parameter τ can be determined empirically and set to τ=5N. Finally, the optimization starts $T^{0}=0$, until the stopping criterion in Equation (20) is reached.

### 3.3. Pseudo-Code

**Figure 4:**
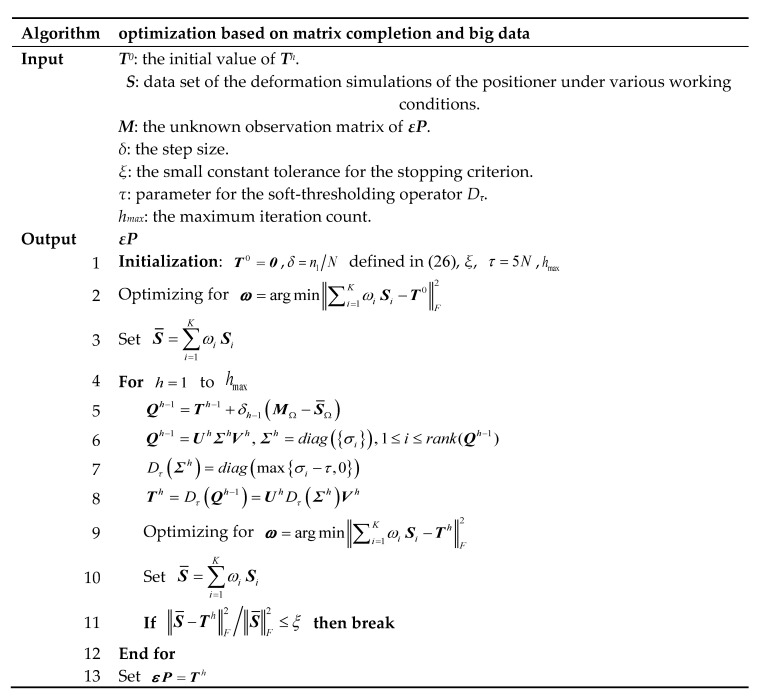
Pseudo-code for the optimization process.

The above optimization process is summarized and the algorithm is given in Figure 4.

## 4. Experiments

**Figure 5:**
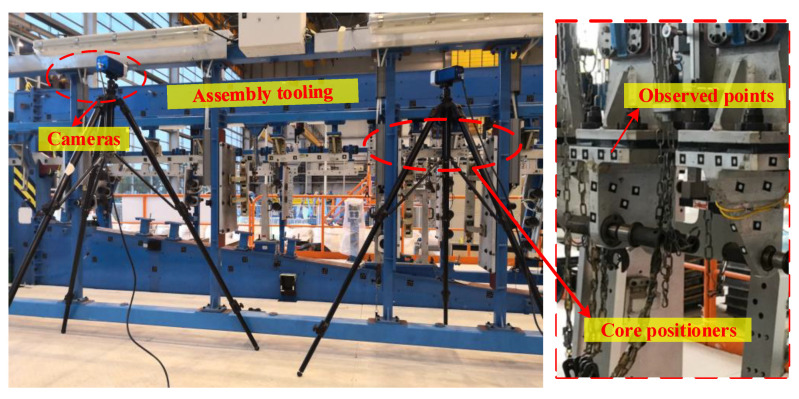
Layout of the facilities.

In the study, the full-field displacement perception experiments of the core positioners, including spar positioners (SPs), actuator positioners (APs) and hinge positioners (HPs) are carried out. To establish the monitoring system, high-accuracy cameras (Chenway, MPS/M20) are adopted to observe the 3D coordinates of the measuring points with an accuracy of 8 μm + 8 μm/m and a high response of 20 Hz. Then, to acquire the big data sets of the full-field displacements under various working conditions according to the production site, a simulation analysis software named COMSOL Multiphysics 5.2 is employed. The overall layout of the facilities is shown in Figure 5.

### 4.1. Simulation for Full-Field Displacement Data Sets

**Figure 6:**
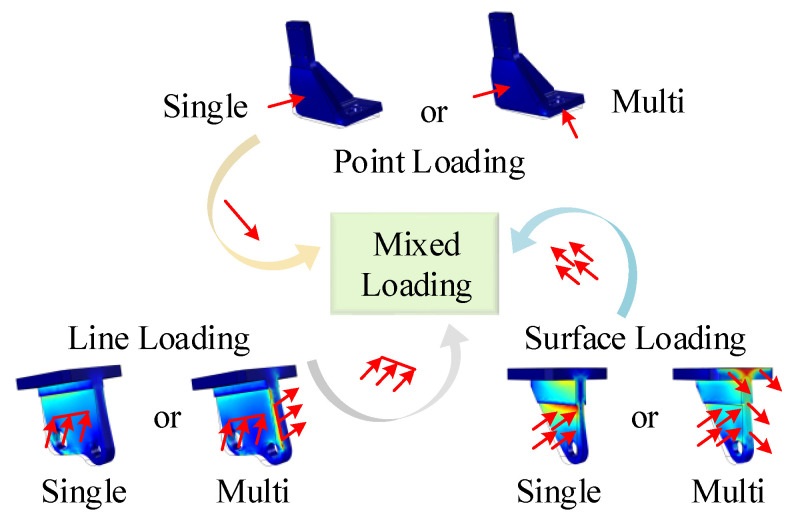
Force loading in the simulation tests.

Simulations tests under various working conditions according to the production site are carried out. Through force analysis during the actual production site, the frequent loading points, lines and surfaces are first obtained. Then, in order to fully consider the various working conditions in the actual production process, the loads on the core components are mainly summarized as four aspects: point loading (including single-point or multi-point loading), line loading (including single-line or multi-line loading), surface loading (including single-surface or multi-surface loading) and mixed loading (including point-line loading, point-surface loading and line-surface loading), which are shown in Figure 6.

According to the actual working conditions, the simulation parameters can be set as: (a) when the point loading is applied, the force amplitude is from −1000 N to +1000 N with a step of 100 N (21 steps in total); (b) when the line loading is applied, the force amplitude is from −1000 N/m to +1000 N/m with a step of 100 N/m (21 steps in total); (c) when the surface loading is applied, the force amplitude is from −1000 N/m^2^ to +1000 N/m^2^ with a step of 100 N/m^2^ (21 steps in total). In consideration of the computer calculation ability, three points, three lines and three surfaces are set on each positioner for point loading, line loading and surface loading, respectively, and the counts of the simulation groups are set as in Table 1.

**Figure 7:**
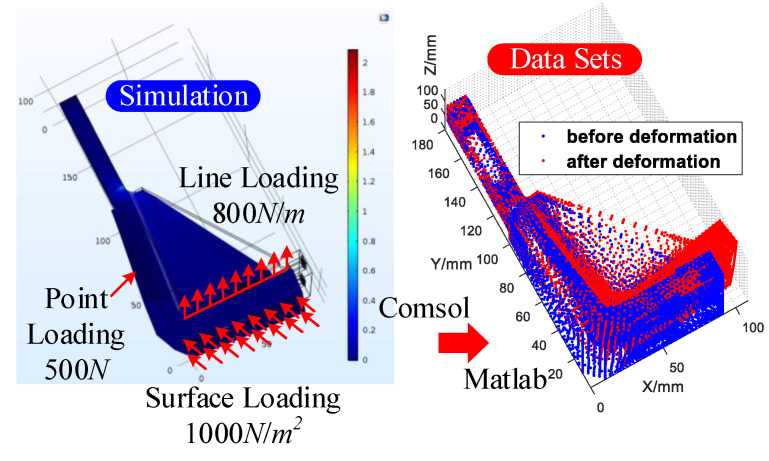
Full-field displacement data set acquisition in the simulation test.

As shown in Table 1, the big data set ***S*** in Equation (11) is finally made up of 293,895 simulation groups in total. Figure 7 shows the full-field displacement data set acquisition process for any set of the simulation tests (the deformation is magnified, and the max value of the deformation is 0.112 mm).

### 4.2. Accuracy Analysis Based on Simulation Data Sets

**Figure 8:**
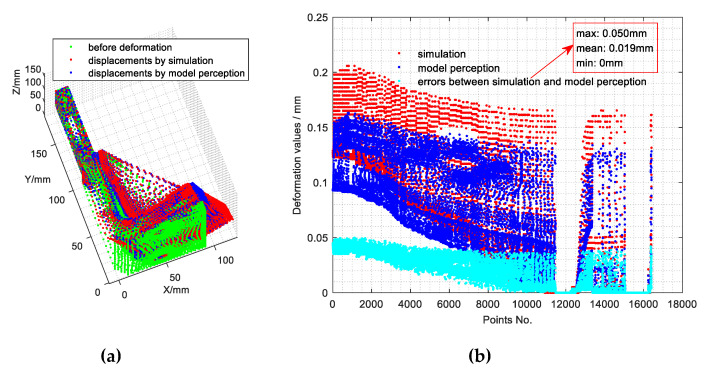
Comparison between the simulation and the model perception. (**a**) Full-field displacements based on simulation and model perception. (**b**) Absolute errors between the results of simulation and model perception.

To verify the accuracy of the proposed method, accuracy analysis based on the simulation data sets is carried out. The big data set ***S*** (with a group count of 293,895) is divided into two parts, one is used for model solving based on the proposed method, defined by ***S****^1^* (4/5 of ***S***, group count: 235,116), and the other one is used for accuracy testing, defined by ***S****^2^* (the other 1/5 of ***S***, group count: 58,779). Based on ***S****^1^*, the full-field displacement perception model can be solved and then, by applying the model to ***S****^2^*, the full-field displacement calculation results of ***S****^2^* can be obtained. Finally, accuracy analysis is achieved by comparing the calculation results and the simulation results. The results of one of the comparison tests of SPs are shown in Figure 8.

It can be seen from Figure 8a that the full-field displacements calculated by the proposed perception model are in good agreement with those obtained by the simulation. Meanwhile, Figure 8b shows the deformation values of both the simulation results and the model perception results, which are the distances between the full-field displacements before and after the deformation of the component.

**Figure 9:**
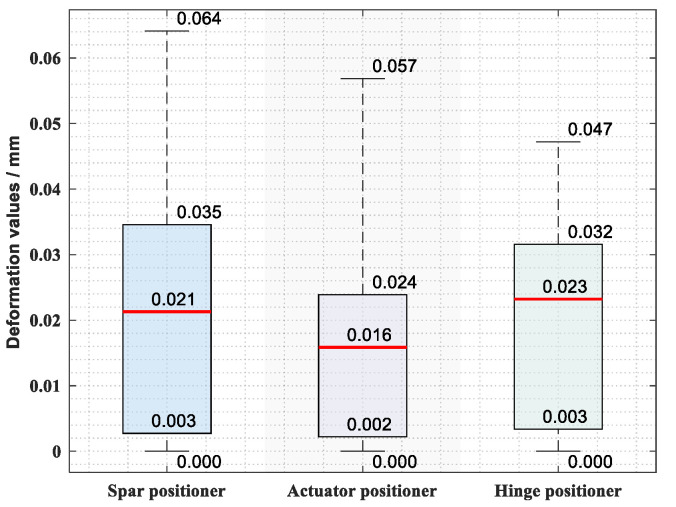
Displacement perception error statistics of all the samples in ***S****^2^*.

Then, for all the comparison tests in ***S****^2^*, the error statistics of the full-field displacement perception results are shown in Figure 9. It can be seen that, by comparison with the simulation, the calculation results based on the proposed displacement perception method can achieve a good precision with the max error less than 0.064 mm and the median error less than 0.023 mm. It indicates that the proposed method can effectively calculate the full-field displacements of a given working condition based on the big data sets of the displacement field of a component under various working conditions.

### 4.3. Digital Twin for the Core Positioners

**Figure 10:**
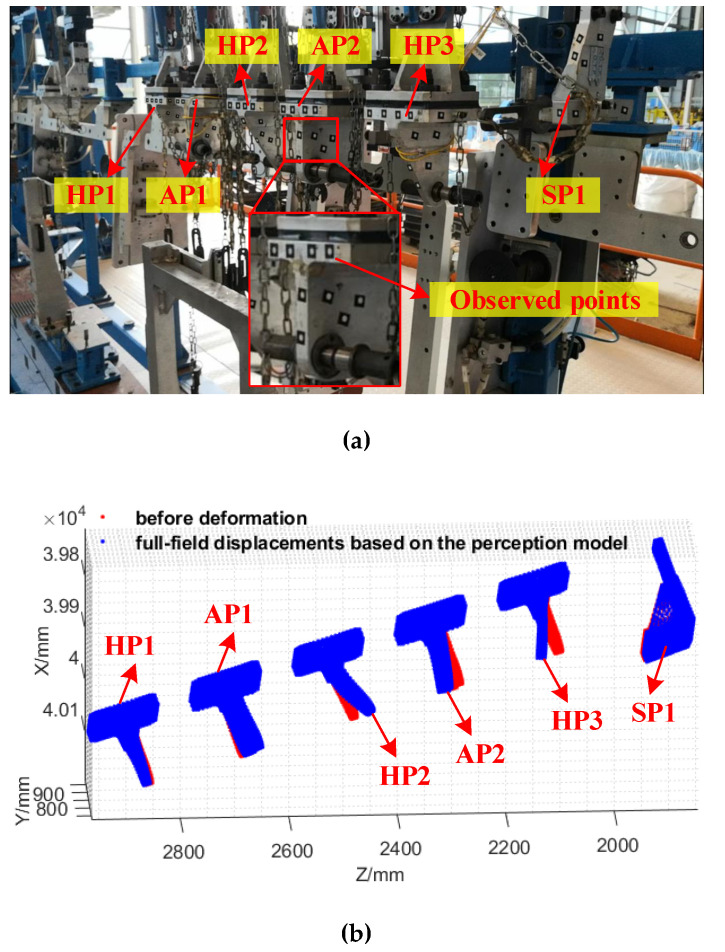
Digital twins for the core positioners in the production site based on the proposed displacement perception method. (**a**) The observed positioners. (**b**) The full-field displacements of the positioners.

After the establishment of the full-field displacement perception and the accuracy tests based on the simulation big data, digital twins for the core positioners in the production site, including SPs, APs and HPs, are created, which are shown in Figure 10.

From Figure 10b, it can be seen that the proposed full-field displacement perception method has a good performance in the production site, which can effectively achieve a digital twin for all the core positioners. Meanwhile, by comparison with the monitoring results of the observed points, the errors of the proposed displacement perception method can be obtained.

**Figure 11:**
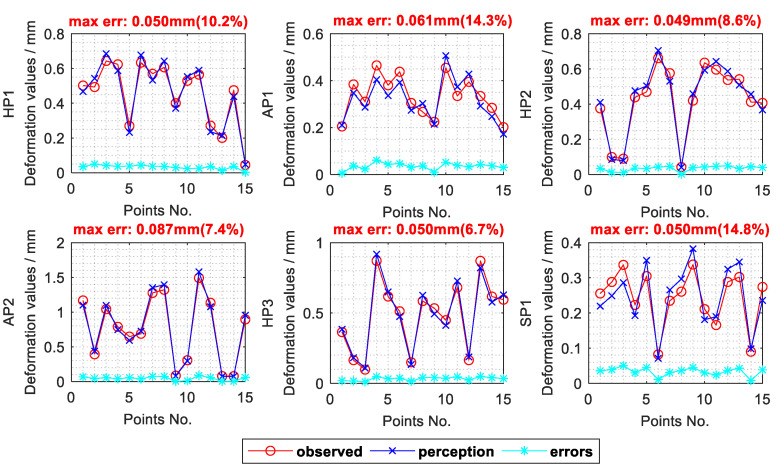
Comparison between the results of actual monitoring and model perception.

Figure 11 shows one of the results from the comparison tests. The red line in each subfigure represents the deformation values of the camera-observed points, that is, the displacement differences of the observed points before and after the deformation. Then, through the proposed full-field displacement perception method, the displacements of the observed points can also be calculated. Similarly, the deformation values of the calculated displacements of the observed points are shown with a blue line. The errors between the observed results and the perception results are shown with a cyan line. It can be seen that the results calculated by the proposed method fit well with the results observed by the cameras, with a max absolute error of less than 0.087 mm and a max relative error of less than 14.8%. In order to better analyze the reliability of the method, repeated experiments are conducted and the errors statistics of all the positioners can finally be achieved.

**Figure 12:**
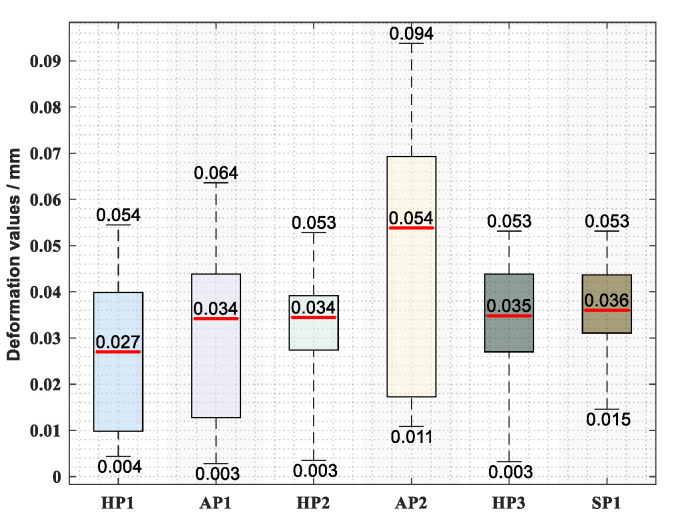
Displacement perception error statistics of all the positioners.

From Figure 12, it can be seen that a good accuracy and stability can be achieved for the full-field displacements calculated by the proposed method. Through repeated experiments in the production site, the max error of the calculated displacements can be less than 0.094 mm, and the median error can be less than 0.054 mm. Meanwhile, a single full-field displacement perception process can be finished in an average timeframe of less than 0.48 s. Thus, the full-field displacements of the core positioners on the assembly tooling can be monitored and calculated precisely in real time by the combination of the cameras and the proposed method, which provide a powerful data basis for the stability of the aircraft manufacturing process.

## 5. Conclusions

In this paper, a real-time full-field displacement perception method with a combination of online multi-point displacement monitoring and matrix completion theory is presented. Full-field displacement perception experiments are conducted on the core components of aircraft assembly tooling, including spar positioners, actuator positioners and hinge positioners. The full-field displacement perception model, based on the observed points and the simulation big data sets, is established. Repeated experiments show that the proposed model is capable of achieving a precise and efficient displacement perception process with a max error of less than 0.094 mm and a median error of less than 0.054 mm in an average timeframe of less than 0.48 s. Meanwhile, full-field displacement perception is an indispensable process in manufacturing, testing and many other fields. With the advantages of high precision and high efficiency, the full-field displacement perception method proposed in this paper can perfectly serve the intelligent manufacturing industries of aircrafts, automobiles, ships, etc.

## Figures and Tables

**Table 1:** Simulation groups of each positioner.

Loading Mode	Group Counts
point loading	$10,647\{\begin{array}{ll} 1\;point: & C_{3}^{1}\times 21^{1}=63\\ 2\;points: & C_{3}^{2}\times 21^{2}=1323\\ 3\;points: & C_{3}^{3}\times 21^{3}=9261 \end{array}$
line loading	10,647
surface loading	10,647
mixed loading	$261,954\{\begin{array}{ll} 1\;point,1\;line: & C_{3}^{1}\times C_{3}^{1}\times 21^{2}=3969\\ 1\;point,1\;surface: & C_{3}^{1}\times C_{3}^{1}\times 21^{2}=3969\\ 1\;line,1\;surface: & C_{3}^{1}\times C_{3}^{1}\times 21^{2}=3969\\ 1\;line,1\;line,1\;surface: & C_{3}^{1}\times C_{3}^{1}\times C_{3}^{1}\times 21^{3}=250,047 \end{array}$
total	293,895
